# The dual-site agonist for human M2 muscarinic receptors Iper-8-naphtalimide induces mitochondrial dysfunction in *Saccharomyces cerevisiae*

**DOI:** 10.15698/mic2025.12.862

**Published:** 2025-12-12

**Authors:** Angela Cirigliano, Antonia Amelina, Elena Passarini, Alessandra Ricelli, Nicole Balasco, Mattia Mori, Bruno Botta, Maria Egle De Stefano, Claudio Papotto, Claudia Guerriero, Ada Maria Tata, Teresa Rinaldi

**Affiliations:** 1 Institute of Molecular Biology and Pathology, CNR c/o Department of Chemistry, Sapienza University of Rome, Piazzale A. Moro 5, 00185 Rome, Italy; 2 Department of Biology and Biotechnologies, Sapienza University of Rome, Piazzale A. Moro 5, 00185 Rome, Italy; 3 Department of Biotechnology, Chemistry and Pharmacy, University of Siena, via Aldo Moro 2, 53100 Siena, Italy; 4 Dipartimento di Chimica e Tecnologie del Farmaco, Sapienza University of Rome, Piazzale A. Moro 5, 00185 Rome, Italy; 5 Research Centre of Neurobiology “Daniel Bovet”, Sapienza University of Rome, 00185 Rome, Italy; 6 Department of Pharmaceutical Sciences, University of Milan, 20133 Milan, Italy

**Keywords:** Glioblastoma, M2 muscarinic receptor, orthosteric agonist, dualsteric agonist, ergosterol, mitochondrial DNA, modeling in *S. cerevisiae*

## Abstract

Glioblastoma is a malignant astrocytic tumor of the brain. A significantly decrease of glioblastoma cell proliferation and survival can be achieved by activating the M2 muscarinic acetylcholine receptor (a G protein-coupled receptor, or GPCR) with two agonist molecules, the orthosteric agonist Arecaidine Propargyl Ester (APE) and the dual-steric agonist Iper-8-naphthalimide (N-8-Iper). In glioblastoma cells, these agonists caused mitochondrial damage and an altered lipid profile. To characterize the mitochondrial dysfunction induced by the muscarinic agonists, we tested APE and N-8-Iper in *S. cerevisiae*, a yeast model system specifically suitable to study the activity of molecules of pharmaceutical interest on mitochondria. N-8-Iper, but not APE, induced mitochondrial dysfunction in *S. cerevisiae* cells in a time- and concentration-dependent manner. These results suggest that the agonist N-8-Iper on glioblastoma cell cultures has a direct effect on mitochondrial function. Moreover, since GPCRs are evolutionarily conserved from yeast to humans, these results confirm that the yeast system is a suitable model for studying human GPCRs.

## INTRODUCTION

The human genome encodes 831 G protein-coupled receptors (GPCRs). Many of these receptors are the subject of research in both basic and applied medical fields and are the target of one third of all drugs approved by the US Food and Drug Administration (FDA) 1.

The human M2 muscarinic acetylcholine receptor (mAChR) is a GPCR, consisting of a monomeric structure with seven transmembrane helices. It is associated with G ${}_{i}$ proteins, which primarily inhibit adenylate cyclase activity, primarily causing a decrease in intracellular levels of cAMP concentrations 2, 3. In cancer cells, embryonic genes can be reactivated during malignant growth, including the mAChR genes 4. The expression of mAChRs in several metastatic and primary cancers is well documented, particularly the expression of the M2 mAChR in glioblastoma, in both stable cell lines and stem cells 5, 6. Previous data obtained in human glioblastoma cells showed that the activation of the M2 muscarinic receptor, with agonist molecules, dramatically reduced the proliferative rate and cell survival of cancer cells, exhibiting cytotoxic effects 7–10 and mitochondrial defects (unpublished results). The antiproliferative effect is more pronounced with the dualsteric agonist Iper-8-naphthalimide (N-8-Iper) than with the orthosteric agonist Arecaidine Propargyl Ester (APE), probably due to N-8-Iper’s higher affinity for and selectivity of the M2 mAChR 7–9; dualsteric agonist is a specific ligand able to bind simultaneously both the orthosteric and allosteric site of the receptor, combining both binding affinity (at the orthosteric site) with a high selectivity (at the allosteric site) 11.

GPCRs have been conserved throughout evolution, from yeast to humans, and the yeast system is considered a useful model for studying human GPCRs 12–15. When expressed in yeast, mammalian GPCRs have been shown to functionally couple to either endogenous yeast G α or co-expressed mammalian G α subunits. They are characterized by similar pharmacology in response to agonists or antagonists as in native cells 16. In *S. cerevisiae*, a single GPCR signaling pathway controls the yeast mating pheromone response 17, 18. Haploid yeast cells (MATa or MAT α ) secrete pheromone peptides ( α -factor) and express the GPCRs Ste2 or Ste3, respectively. During mating, Ste2 or Ste3, which are activated by the peptide secreted by the opposite cell type, activate the heterotrimeric G protein signal cascade, leading to mating. Recently, the three-dimensional structure of the GPCR Ste2 was experimentally determined 19, 20.

To gain insight into the impaired mitochondrial function observed in glioblastoma cells treated with the M2 agonists, we treated *Saccharomyces cerevisiae* yeast cells with the M2 mAChR agonists. *S. cerevisiae* is an excellent unicellular model that can survive without mitochondrial function by utilizing fermentable carbon sources, a unique feature among eukaryotes. *S. cerevisiae* is also a validated model for studying human and mitochondrial diseases, as more than 30% of the genes implicated in human disease have orthologues in yeast 21–26. Furthermore, *S. cerevisiae* is a suitable model for investigating the activity of molecules of pharmaceutical interest 27–29.

The results showed that N-8-Iper, but not APE, induced the loss of mitochondrial DNA and consequently a block of respiration in yeast, confirming that mitochondria are one of the first targets of N-8-Iper signaling.

## RESULTS

### Molecular docking of APE and N-8-Iper with the Ste2 yeast receptor: a structural view of the protein-ligand interactions

**Figure 1:**
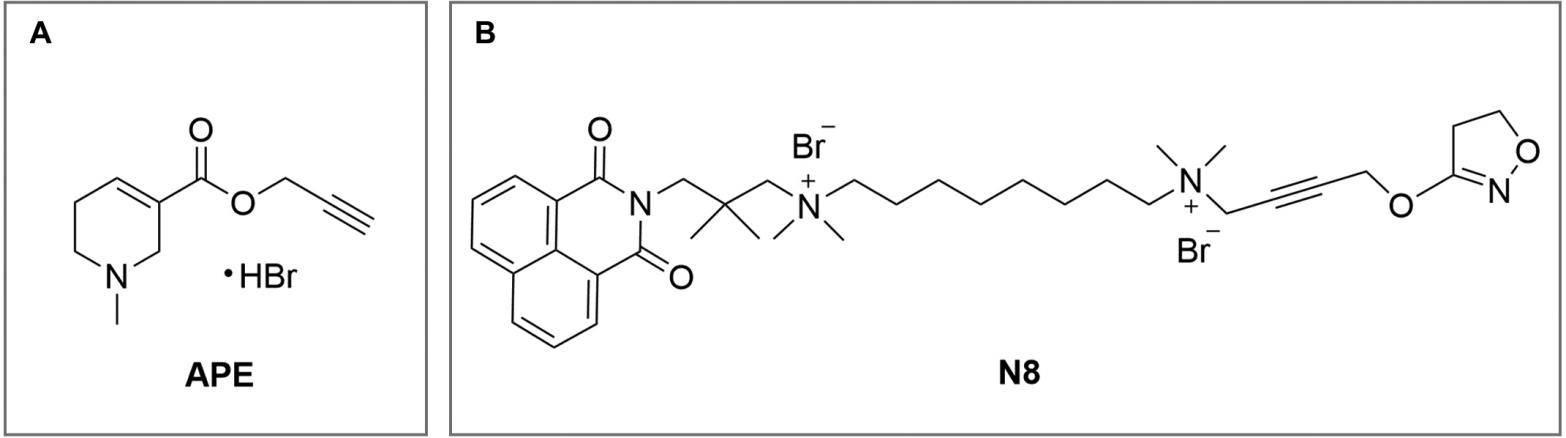
Molecular structures of the two ligands investigated in this study. **(A)** Arecaidine Propargyl Ester (APE). **(B)** Iper-8-naphtalimide (N8).

In order to evaluate yeast cells as a model for studying the mitochondrial effects of APE and N-8-Iper (Figure 1 **A**–**B**), which are characterised as agonists of the M2 muscarinic receptor subtype (UniProtKB P08172), we initially compared the structures of the M2 muscarinic receptor (PDB ID: 7T90) and its yeast homologue, the pheromone alpha-factor receptor Ste2 (UniProtKB D6VTK4; PDB ID: 7AD3).

**Figure 2:**
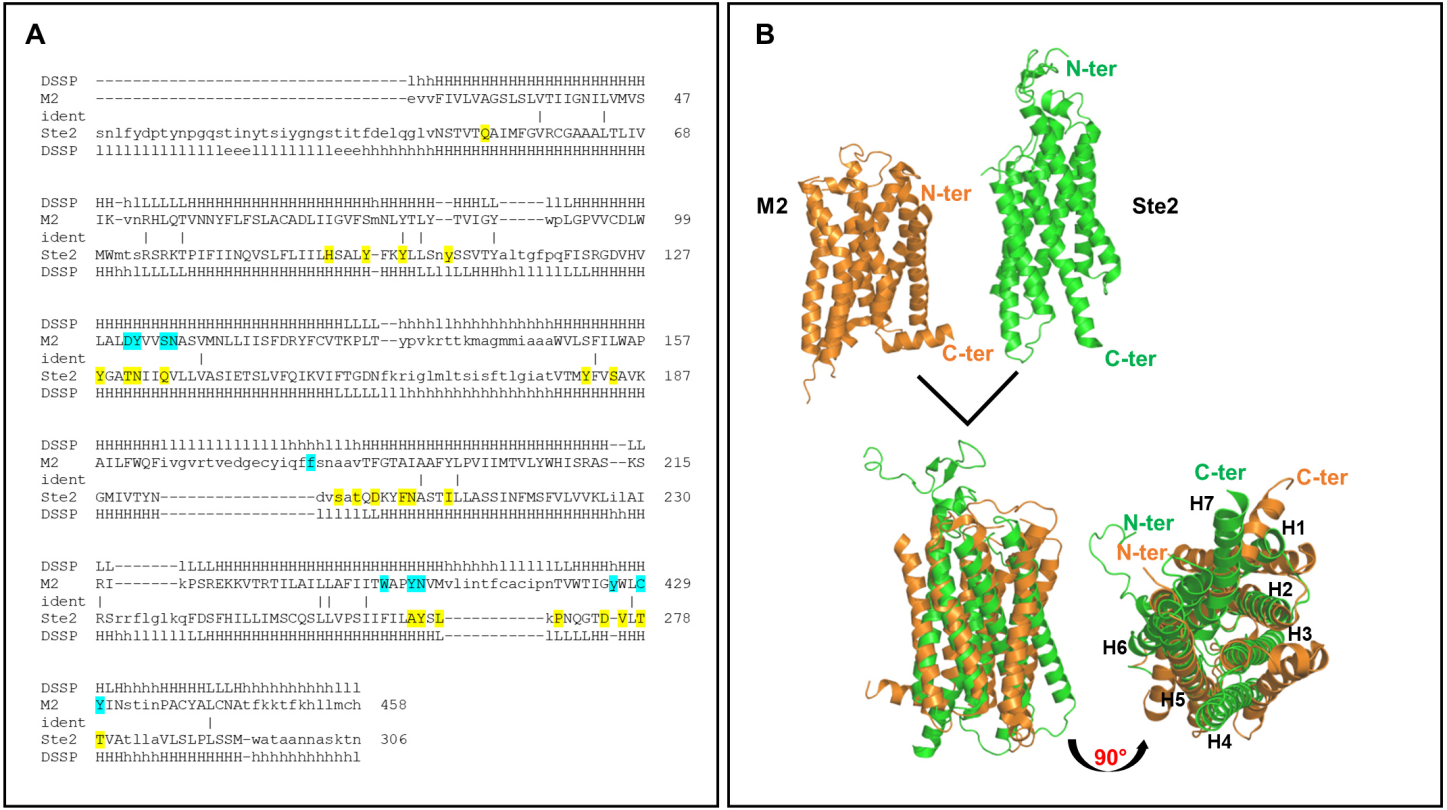
Sequence alignment and structural superimposition. **(A, B)** Sequence alignment (A) and structural superimposition (B) between M2 muscarinic receptor (orange, PDB ID: 7T90) and Ste2 (green, PDB ID: 7AD3) performed using the Dali server (http://ekhidna2.biocenter.helsinki.fi/dali/). M2 muscarinic receptor and Ste2 residues involved in interactions with their respective natural ligand (acetylcholine for M2 muscarinic receptor and α -factor peptide for Ste2) are highlighted in cyan and yellow, respectively.

A structural comparison of these models using the Dali server 30 revealed that, despite belonging to the GPCR family, the two proteins shared only 9% amino acid sequence identity in the superimposed region (Figure 2 **A**). However, the comparison of the experimental models revealed that they exhibited the same protein folding, with a transmembrane channel made of seven α -helices (H1-H7, Figure 2 **B**). Inspection of the superimposed models reveals that the H2, H3 and H5 helices are optimally aligned. Although slight displacements are observed for the other helices, the overall structural organisation remains well preserved (Figure 2 **B**). It is also worth noting that the regions of the proteins responsible for recognising their respective natural ligands (acetylcholine (ACh) for the M2 muscarinic receptor and the α -factor peptide for Ste2) partly overlap (Figure 2 **A**).

**Figure 3:**
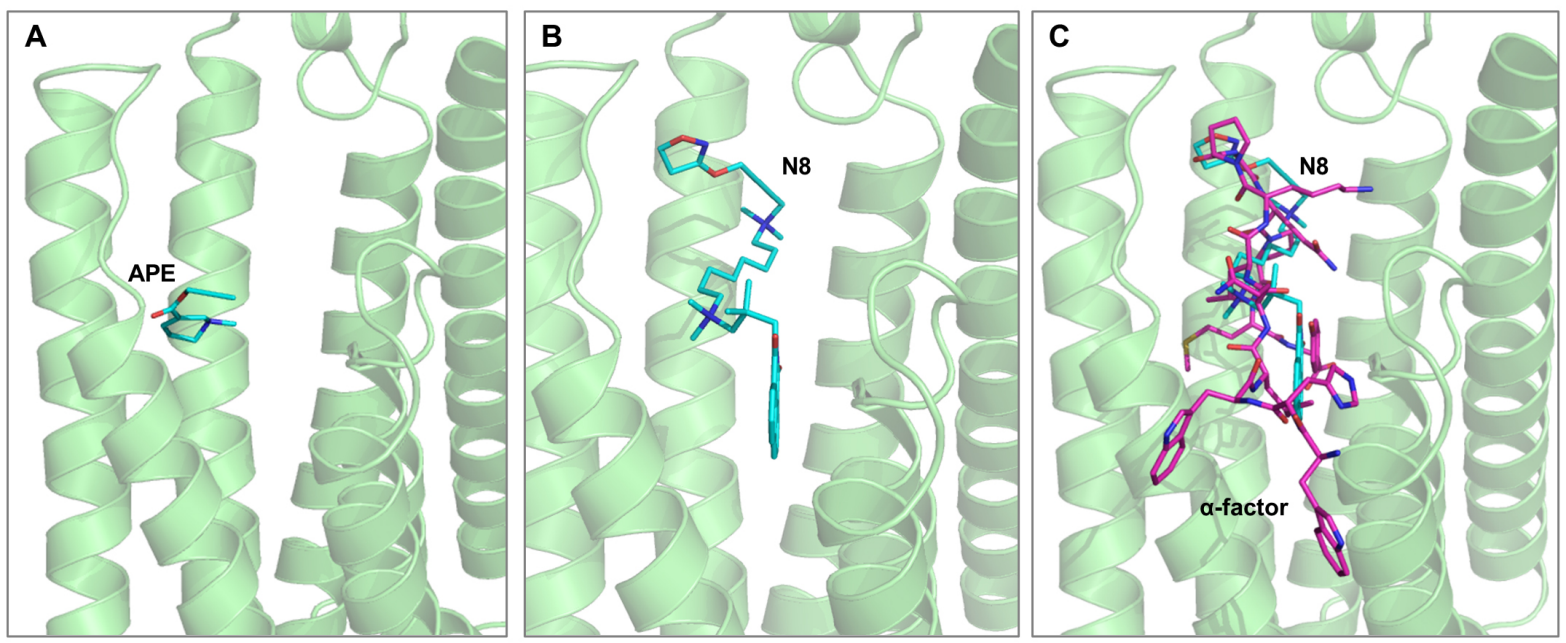
Predictive binding mode. **(A-C)** Predictive binding mode of APE **(A)** and N-8-Iper (N8) **(B)** on *S. cerevisiae* Ste2 protein and comparison with the interaction of the natural ligand, the α -factor peptide **(C)**. APE and N-8-Iper are shown as cyan sticks. Ste2 and its ligand (PDB ID: 7AD3) are shown as green cartoon and magenta sticks, respectively.

These results prompted us to use molecular docking simulations to assess the binding of human M2 mAChR agonists to the Ste2 yeast receptor homologue. The Ste2-APE and Ste2-N-8-Iper complexes were generated using molecular docking (Figure 3 **A**–**B**). The predicted models of the protein-ligand complexes were examined in detail from a structural point of view. The docking pose of N-8-Iper showed excellent occupancy of the Ste2 binding site (Figure 3 **B**), demonstrating remarkable shape complementarity and overlap with the binding mode of the physiological ligand (Figure 3 **C**) as determined experimentally (PDB ID: 7AD3) 19.

**Figure 4:**
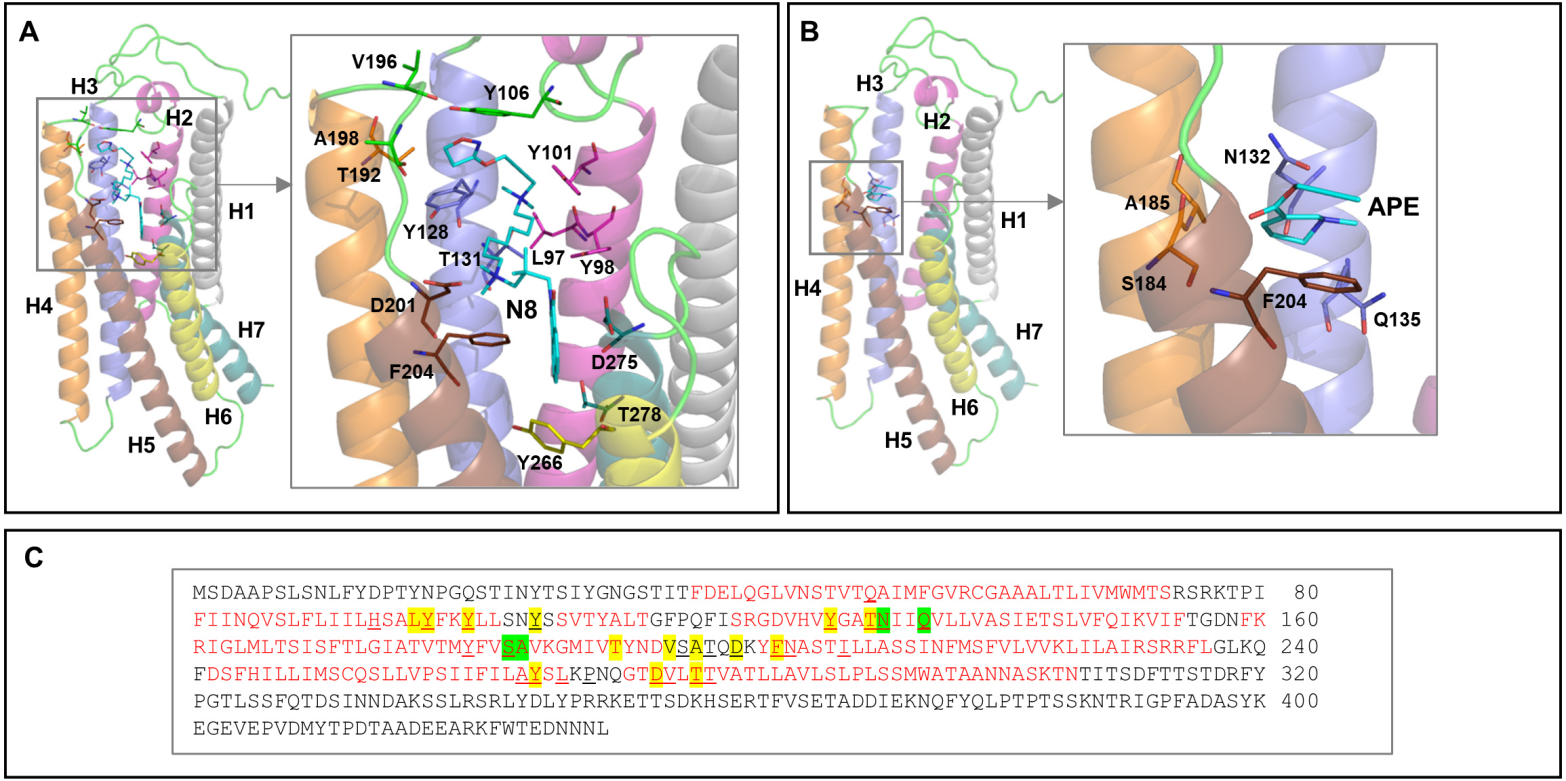
Model of Ste2-N8-Iperand Ste2-APE complexes. **(A, B)** Model of Ste2-N8-Iper **(A)** Ste2-APE **(B)** complexes: residues of Ste2 interacting with the ligand are shown as sticks. Amino acid sequence of Pheromone alpha factor receptor Ste2 (UniProtKB D6VTK4) from *Saccharomyces cerevisiae* **(C)**. The seven helices that form the transmembrane domain are in red. Residues of Ste2 that interact with N-8-Iper and APE are highlighted in yellow and green, respectively. Phe204 forms contacts with both ligands. Residues of Ste2 that interact with the α -factor peptide are underlined.

N-8-Iper is primarily stabilised by van der Waals interactions with the aliphatic/aromatic side chains of Ste2 amino acid residues. As shown in Figure 4 **A**, the residues involved in these contacts belong to all helices of the receptor except H1. The extended structure of N-8-Iper enables it to be properly placed in the protein’s binding site, where it establishes a significant number of interactions with the ligand. Conversely, the smaller size of APE prevents it from occupying the entire protein site designated for binding the α -factor peptide (Figure 3). Only part of the molecule interacts with three of the seven helices that form the receptor domain (H3, H4 and H5) (Figure 4 **B**). Interestingly, in both cases, most of the interacting residues (Tyr98, Tyr101, Tyr106, Tyr128, Thr131, Asp201, Phe204, Tyr266, Asp275 and Thr278 for Ste2-N8-Iper, and Asn132, Gln135, Ser184 and Phe204 for Ste2-APE) are the same as those involved in binding the α -factor peptide, as revealed by the experimental structure (PDB ID: 7AD3) (Figure 4 **C**). Although N-8-Iper is expected to interact more effectively with Ste2, these results suggest that both molecules can be accommodated in the Ste2 binding site, thereby affecting its biological functions. These theoretical results suggest that yeast could be a reliable model for the *in vivo* testing of APE and N-8-Iper. Our yeast-based findings also indirectly imply that Ste2 may share an evolutionary origin with the human GPCR receptor family.

### Effects of the selected mAChR agonists on yeast cells

**Figure 5:**
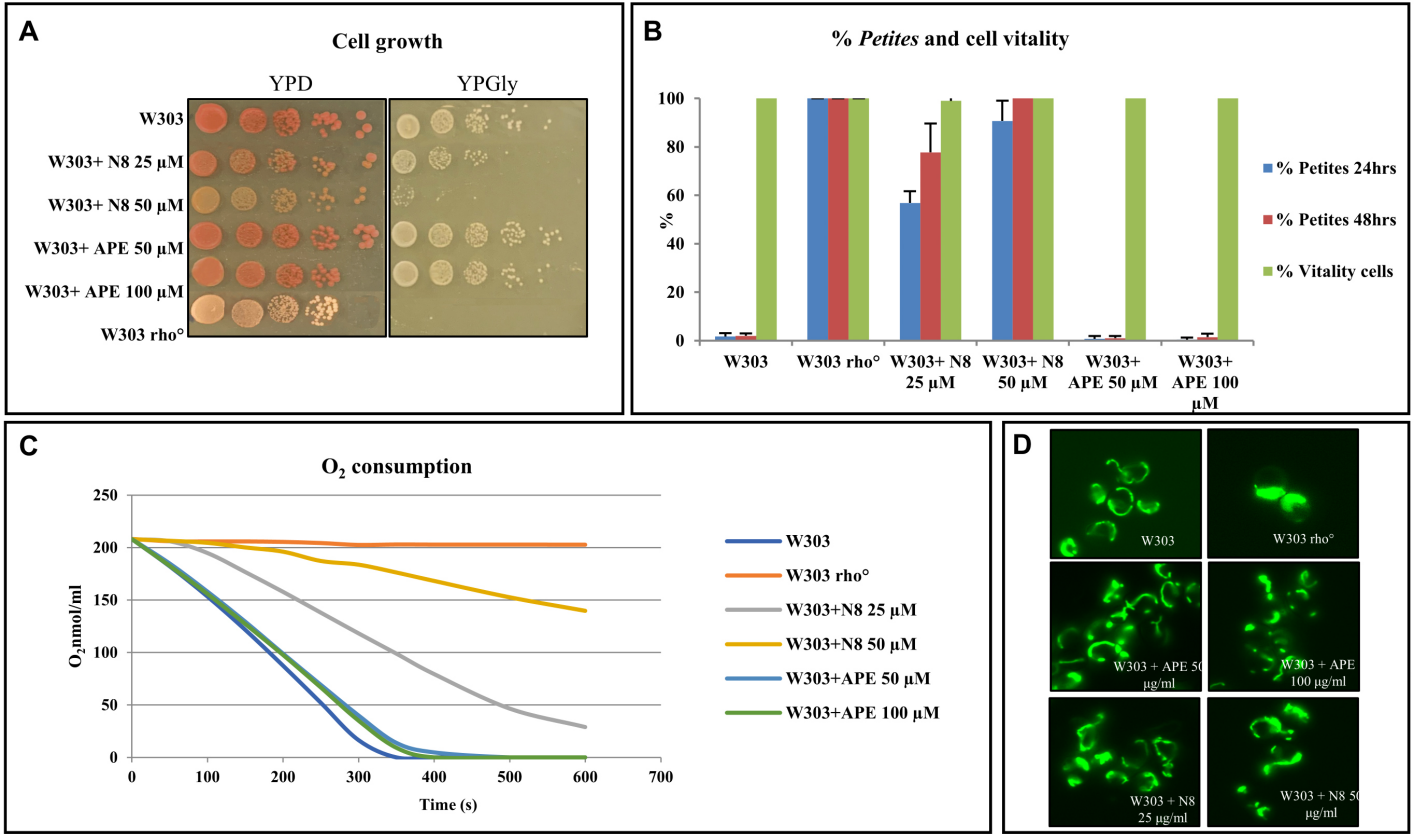
The muscarinic receptor agonist N-8-Iper (N8) induces a mitochondrial defect in the W303*S. cerevisiae*yeast cells. ** (A) ** Growth of yeast cells treated with muscarinic agonists in complete medium (YPD) and in a respiratory carbon source (YPGly), the agonist N-8-Iper impairs the growth in *S. cerevisiae* at the concentration of 50 μ M*,* pictures taken after 24 hours of growth, while APE has no effect on yeast cells growth. **(B)** The muscarinic receptor agonist N-8-Iper induces a *petites* phenotype in *S. cerevisiae* and impairs respiration. The histogram shows the % of *petites* production in yeast cells treated with the muscarinic agonists. N-8-Iper, but not APE, induces *petites* production. The vitality of the yeast cells is not affected by agonists treatment. The W303 rho ${}^{\circ }$ cells (devoided of mitochondrial DNA and defective in the mitochondrial respiration) was used as a control. **(C)** The respiration rate of yeast cells treated with N-8-Iper is impaired compared to the wild type strain, APE has no effect on the respiration rate. The oxygen consumption rate of W303, W303 rho ${}^{\circ }$ and W303 cells treated with agonists is expressed in O ${}_{2}$ nmol/ml. **(D)** The loss of mitochondrial function in yeast cells treated with N-8-Iper is coupled with a collapsed mitochondrial phenotype. Mitochondrial morphology was visualised with a Gfp targetted into the mitochondria in exponential phase.

A broad range of APE and N-8-Iper concentrations were tested in yeast cells (data not shown) to determine the concentrations to be used in the experiments. 100 μ M and 50 μ M for APE and 50 μ M and 25 μ M for N-8-Iper, respectively. The cells were grown overnight in the presence of the agonists and then spotted onto plates containing a fermentable (glucose, YPD) or respirable (glycerol, YPGly) carbon source (Figure 5 **A**). On YPD plates, yeast cells treated with both agonists (APE and N-8-Iper) exhibited a growth rate similar to that of untreated cells. This suggests that the treatment with agonists did not affect cell viability. Growth tests in YPGly produced different results; plates containing only glycerol are typically employed to assess mitochondrial function in yeast, since glycerol is metabolised in the mitochondria. W303 rho ${}^{\circ }$ cells, which lack mitochondrial DNA and have defective mitochondrial respiration, were used as a control as they do not grow in YPGly. Growth on glycerol was absent in cells treated with N-8-Iper only, indicating specific impairment of mitochondrial function, and this phenotype was N-8-Iper concentration-dependent. The lack of growth in YPGly could be due to a loss of mitochondrial DNA in the cells. Thus, a test for *petites* production was performed (Figure 5 **B**–S1) 31, 32.

Following 24 and 48 hours of cell growth with agonist treatment, APE did not produce *petites*. In contrast, 100% of *petites* were produced in cells treated with 50 μ M N-8-Iper after 48 hours. Production of *petites* in cells treated with the N-8-Iper agonist was both time- and dose-dependent. *Petite* colonies were visualised using a fluorescence microscope after DAPI staining, revealing that *petite* colonies are formed by rho ${}^{\circ }$ cells (cells that have completely lost mitochondrial DNA). Finally, to verify the respiration rate of the cells, we measured the oxygen consumption of the treated cells (Figure 5 **C**). The respiratory rate was impaired only in the wild-type strain treated with the N-8-Iper agonist.

To validate the results, we repeated the experiments using the BY4741 yeast strain, which has a different genetic background to the W303 strain that was used as the reference strain in this study. Both the W303 and BY4741 wild-type strains are derived from the S288C strain. However, BY4741 has a mutation in the HAP1 gene, which regulates several genes involved in electron transfer reactions in response to heme 33. We proceeded to test the agonists at the same concentrations used with the W303 strain, and the results confirmed the previous observations in the BY4741 genetic background (Figure S2). Only slight differences were observed: in the W303 strain, treatment with 50 μ M N-8-Iper induced 100% petites after 48 hours of growth, whereas in the BY4741 strain, the percentage of petites was 80%. BY4741 also exhibited reduced vitality compared to the W303 strain, however this phenotype may be attributed to the impaired function of the HAP1 gene in BY4741. Overall, the results suggest that both wild-type strains exhibited a mitochondrial dysfunction defect when treated with N-8-Iper, whereas treatment with APE did not induce mitochondrial dysfunction in yeast cells. To verify that the molecular target of N-8-Iper is the Ste2 receptor, a STE2 gene deletion strain in a BY 4741 Mat a genetic context was treated with the agonist N-8-Iper, and the results showed that the mitochondrial defects caused by N-8-Iper were absent when the Ste2 receptor was not present. This suggests that the M2 agonist is specific to the Ste2 receptor (Figure S2D).

### The effects of selected mAChR agonists on yeast mitochondria

The absence of mitochondrial DNA can cause defects in mitochondrial morphology, resulting in collapsed mitochondria 34, 35. Therefore, we transformed the wild-type strain with a plasmid containing mtGFP to visualize mitochondrial morphology in cells during the exponential growth phase. Cells treated with N-8-Iper showed mitochondrial morphological defects, including aggregated and collapsed mitochondria. This phenotype is characteristic of rho ${}^{\circ }$ cells (Figure 5 **D**).

### Effects of the selected mAChR agonists on ergosterol content in yeast cells

**Figure 6:**
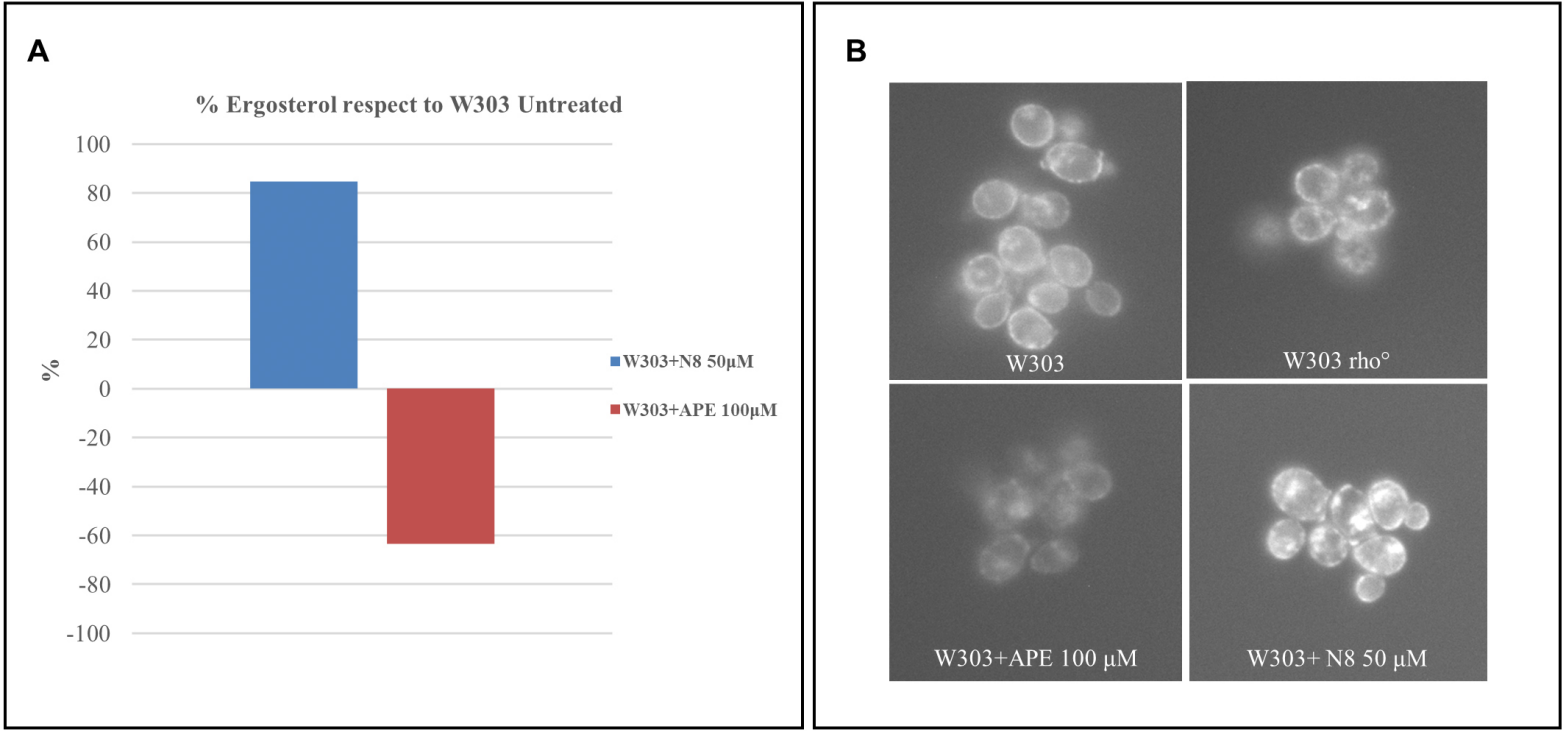
The yeast ergosterol content is altered with the muscarinic agonist treatment. **(A)** HPLC analysis showed that ergosterol is less abundant in yeast cells treated with APE and more abundant in cells treated with N-8-Iper (N8), compared to the not treated wild-type strain. **(B)** *In vivo* Filipin staining of cell cultures treated with APE and N-8-Iper agonists. The fluorescence intensity of the filipin, a fluorescent sterol binding dye, is directly proportional to the ergosterol content in plasma membrane.

We also tested whether M2 agonists could modify the content of ergosterol in yeast. High-performance liquid chromatography (HPLC) analysis showed that ergosterol was less abundant in cells treated with APE and more abundant in cells treated with N-8-Iper, compared to the wild-type strain (Figure 6 **A**). As specialized sterol-rich membrane domains can be visualised in yeast using Filipin, a fluorescent sterol-binding dye 36, we visualised the Filipin staining of cell cultures treated with the agonists APE and N-8-Iper *in vivo*. Measuring the fluorescence intensity of Filipin, which is directly proportional to the ergosterol content of the plasma membrane, confirmed the higher and lower sterol content in cells treated with N-8-Iper and APE, respectively (Figure 6 **B**).

These results are consistent with the observation that N-8-Iper impairs mitochondrial function and causes lipid accumulation in GB cells (unpublished results).

## DISCUSSION

In this study, we tested two agonists of the human M2 muscarinic receptor subtype, APE and N-8-Iper, in yeast cells. These molecules were selected for their antiproliferative activity against glioblastoma cell lines (unpublished results). Treatment with APE did not result in a significant yeast phenotype, whereas treatment with N-8-Iper produced detrimental effects on mitochondrial function, inducing *petites* formation and impairing respiratory function. These results were validated using another wild-type strain of *S. cerevisiae* (BY4741). Furthermore, the STE2-deleted strain treated with N-8-Iper did not exhibit mitochondrial defects, suggesting that the M2 agonist is specific to the Ste2 receptor.

The mitochondrial defects observed in cells treated with the muscarinic agonist were accompanied by abnormal mitochondrial morphology; the treated cells exhibited aggregated and collapsed mitochondria. These findings are of great interest, given that the glioblastoma cell line treated with N-8-Iper showed alterations that could be associated with mitochondrial defects and oxidative stress, including cytotoxic and genotoxic effects 7. The results obtained using the yeast model suggest that muscarinic agonists could act on the mitochondrial pathway of glioblastoma cell lines, thereby promoting an autophagic and/or apoptotic process 9. Thus, the yeast system once again validated a mitochondrial phenotype, offering insight into future experiments on the glioblastoma cell line. The mitochondrial damage could be considered a direct consequence of N-8-Iper activity. The Ste2 receptor may accommodate a non-native ligand such as N-8-Iper and still be able to transduce a signal, resulting in mitochondrial dysfunction. Although we did not test direct contact between N-8-Iper and the Ste2 receptor, molecular docking predicted that only N-8-Iper would be active in yeast as it occupies the receptor site in a manner similar to that of the natural ligand. Consistent with this prediction, only N-8-Iper induced specific mitochondrial dysfunction in yeast in a time- and concentration-dependent manner.

Interestingly, in a screening to identify yeast nuclear genes indispensable for respiratory growth, several mutants required for mating were selected due to impaired expression and maintenance of the mitochondrial genome. These mutants include Δ aga2, Δ erg6, Δ htl1, Δ sst2, Δ ste2, Δ ste3 and Δ ste20 37. Further investigation of the involvement of these genes in mitochondrial function is warranted.

## MATERIALS AND METHODS

### Molecular modelling

The 3.30 Å resolution cryo-electron microscopy (cryo-EM) structure of the *Saccharomyces cerevisiae* Ste2 dimer coupled with G proteins was used as a rigid receptor in molecular docking simulations (PDB ID: 7AD3) 19. Small molecules were prepared for docking with the Omega application version 3.1.0.3 (OpenEye, Cadence Molecular Sciences, Santa Fe, NM http://www.eyesopen.com) 38. Docking simulations were performed with FRED version 3.3.0.3 (OpenEye, Cadence Molecular Sciences, Santa Fe, NM) using default parameters and the highest docking accuracy 39. The cryo-EM model of the ACh-bound M2 receptor (PDB ID 7T90) was used for the structural comparison performed with the Dali server (http://ekhidna2.biocenter.helsinki.fi/dali/) 30. Figures of structural models were created using PyMOL molecular visualization program (https://pymol.org/).

### Yeast strains and growth conditions

The yeast strains used in this study were the *S. cerevisiae* W303-1A (MAT a, his3–11, ade2–1, leu2–3, − 112, ura3–1, trp1–1, can1–100), BY4741 (MAT a, his3 Δ , leu2 Δ , met15 Δ , ura3 Δ ) and Δ Ste2 (MAT a, ura3 Δ 0, leu2 Δ 0, his3 Δ 1, met15 Δ 0, YFL026w :: kanMX4) (EUROSCARF). Yeast culture media: YPD (1% bacto peptone, 1% yeast extract and 2% glucose), was used as a rich medium and YPGly (1% bacto peptone, 1% yeast extract and 3% glycerol) was used to demonstrate respiration in yeast colonies. 2.3% bacto agar (Difco) was added to obtain a solid media.

### Treatment of cells with N-8-Iper and APE

Arecaidine Propargyl Ester hydrochloride (APE, Sigma-Aldrich, Milan, Italy) is a synthetic alkaloid obtained by modifying arecaidine, which is a natural alkaloid derived from *Areca nut*. Iper-8-naphthalimide (N-8-Iper) was synthesised at the University of Milan according to a published protocol 40. These ligands have been proven to selectively bind to the M2 mAChR subtype through pharmacological binding and M2 mAChR knockdown experiments 5, 7. M2 mAChR agonists were dissolved in distilled water.

*S. cerevisiae* cells were grown in YPD until they reached stationary phase. Then, 10 ${}^{4}$ cells/mL were inoculated into YPD and YPD supplemented with APE at concentrations of 100 μ M and 50 μ M, and with N-8-Iper at concentrations of 50 μ M and 25 μ M. The cultures were then incubated at 28 ${}^{\circ }$ C for 24 hours until a density of 1 × 10 ${}^{7}$ cells/mL was reached. These concentrations were chosen based on those found to be effective in human glioblastoma cells 5, 9 and following a preliminary assessment of a range of concentrations from 10 to 200 μ M (data not shown). The treated cells were then used for several assays.

### Spot assay of yeast cells

The cells were grown in YPD and YPD supplemented with M2 muscarinic receptor agonists at 28 ${}^{\circ }$ C for 24 hours. They were then counted using a Bürker chamber. The cultures were then serially diluted (1  × 10 ${}^{7}$ , 10 ${}^{6}$ , 10 ${}^{5}$ , 10 ${}^{4}$ , 10 ${}^{3}$ cells/mL) and then 5 μ L of each dilution was spotted on YPD and YPGly medium and incubated at 28 ${}^{\circ }$ C for two days.

### Petites assay

The cells were grown in YPD and YPD supplemented with M2 muscarinic receptor agonists until the stationary phase. At the concentration of 1  × 10 ${}^{7}$ cells/mL, 100 cells were spotted on YPD plates and incubated at 28 ${}^{\circ }$ C. After two days, the colonies were replicated on YPGly plates. The rho ${}^{\circ }$ cells were confirmed both by the absence of growth of colonies replicated on YPGly plates (a non-fermentable carbon source) and by the absence of mtDNA, as determined by staining with 4 ${}^{\prime }$ ,6-diamidino-2-phenylindole (DAPI; Sigma-Aldrich).

### Microscopy

The cells were observed using a Zeiss Axio Imager Z1 Fluorescence Microscope with AxioVision 4.8 Digital Image Processing System, the objective lens used was 63 × Oil. Filter sets: 38HE (GFP), 43HE (DsRed). Filters for GFP (470/40 nm excitation and 525/50 nm emission) and DAPI (365 nm excitation and 445/450-nm emission), were used Metamorph software (Universal Imaging, West Chester, PA) was used to deconvolute Z-series and treat the images. To visualise mitochondria, yeast cells were transformed with the pVT100U-mitoGFP plasmid 41. To visualise sterols, yeast cells were treated with Filipin (Filipin III from *Streptomyces filipinensis* – Sigma-Aldrich), a polyene antibiotic that forms specific complexes with free 3- β -hydroxysterols. The fluorescent probe Filipin, with excitation at 360 nm, produces strong fluorescence with an emission maximum at 480 nm 42. Filipin was dissolved in dimethyl sulfoxide (DMSO) and used at a final concentration of 5 μ g/mL. Filipin was added to the medium to stain the cells, which were then observed immediately.

### $O_{2}$ consumption measurement

Respiration studies were performed using a Clark oxygen electrode (Hansatech Instruments) following the methodology outlined by De Luca et al. (2009) 43. The cells were collected at the concentration of 1 × 10 ${}^{8}$ cells/mL and washed with 1 mL of sterile water. The cells were then suspended in 1 mL of sodium phosphate buffer solution (10 mM pH 7.4 containing 4 g/L glucose) and transferred to the reaction vessel of the previously calibrated oxygen electrode.

### Ergosterol extraction

The evaluation of the ergosterol amounts in yeast cells was performed as previously described with minor changes 44. For each yeast strain, 20 OD ${}_{600}$ units of culture grown overnight were harvested. These were then collected into a 2 ml screw cap tube and washed once with sterile distilled water. The cell pellet was then suspended in 600 μ L of an ethanol/KOH solution (2.5 g KOH in 3.6 ml DW, with ethanol added up to 10 ml). The suspension was then incubated in a 88 ${}^{\circ }$ C heat block under constant shaking for two hours. Next, 600 μ L of *n*-heptane and 55 μ L of distilled water were serially added to the mixture, followed by vigorous vortexing for three minutes. The sample was then left to separate into phases. The upper phase was transferred to a new tube, evaporated, and the sample was resuspended in MeOH for analysis in HPLC.

### High Performance Liquid Chromatography (HPLC) analysis

The quantification of ergosterol extracted from yeast cells as described above was performed using HPLC-UV/diode array detector (DAD). The liquid chromatography system (Agilent 1260 Infinity, Agilent Technologies, CA, USA) was equipped with a quaternary gradient pump, an autosampler injection system, a column oven set at 25 ${}^{\circ }$ C, a DAD detector, and a chromatography data system (ChemStation version C.01. 07). A Poroshell-120 C18 column (4.6  × 50 mm, 2.7 μ m particle size, Agilent Technologies, CA, USA) was used. The mobile phase was an isocratic mixture of methanol (MeOH) and water (98:1 v/v) eluting at a flow rate of 1.0 mL/min over 10 minutes. At the end of each analysis, the column was washed for 10 minutes using the same isocratic mixture that was used for the ergosterol analysis. Ergosterol was identified in the cell extracts by comparing the retention time and the UV spectrum of the peak areas recorded in the chromatogram with those of the authentic standard. UV spectra were recorded in the range of 200 to 450 nm. Ergosterol quantification was performed using the external standard method by integrating the peak areas acquired at the wavelength corresponding to its maximum absorbance (280 nm), at the retention time of the corresponding ergosterol standard. Working solutions of ergosterol were prepared in amber glass at a concentration of 50 ng/mL and, by means of a 10-fold dilution, at a concentration of 5 ng/mL in MeOH. After use, the solutions were stored at −25 ${}^{\circ }$ C.

## AUTHOR CONTRIBUTIONS

A.C., A.A., E.P., A.R., N.B., M.M., M.D.S. and C.G. conducted the experiments, B.B, C.P. A.M.T. revised the manuscript, T.R. designed the experiments and wrote the paper.

## SUPPLEMENTAL MATERIAL

All supplemental data for this article are available online at www.microbialcell.com. 


## CONFLICT OF INTEREST

The authors declare no conflicts of interest.

## ABBREVIATIONS

Ach – acetylcholine

APE – arecoidine propargyl ester

GPCR – G-protein-coupled receptor

mACHR – muscarinic acetylcholine receptor

N-8-Iper – Iper-8-napthalimide
