# Supplementary material for: The dual-site agonist for human M2 muscarinic receptors Iper-8-naphtalimide induces mitochondrial dysfunction in Saccharomyces cerevisiae
Source: Microb Cell. 2025 Dec 12;12:290–8. doi: 10.15698/mic2025.12.862 (PMC12713414; doi:10.15698/mic2025.12.862)
Supplement: Supplementary file 1 [file mic-12-290-s01.pdf]

Supplementary Material

The dual-site agonist for human M2 muscarinic receptors Iper-8-naphtalimide induces mitochondrial dysfunction in *Saccharomyces cerevisiae*.

Angela Cirigliano[1,#], Antonia Amelina[2,#], Elena Passarini[2], Alessandra Ricelli[1], Nicole Balasco[1], Mattia Mori[3], Bruno Botta[4], Maria Egle De Stefano[2,5], Claudio Papotto[6], Claudia Guerriero[2], Ada Maria Tata[2,5] and Teresa Rinaldi[2,*]

* Corresponding author: Piazzale A. Moro 5, 00185 Rome, Italy; E-mail address: teresa.rinaldi@uniroma1.it

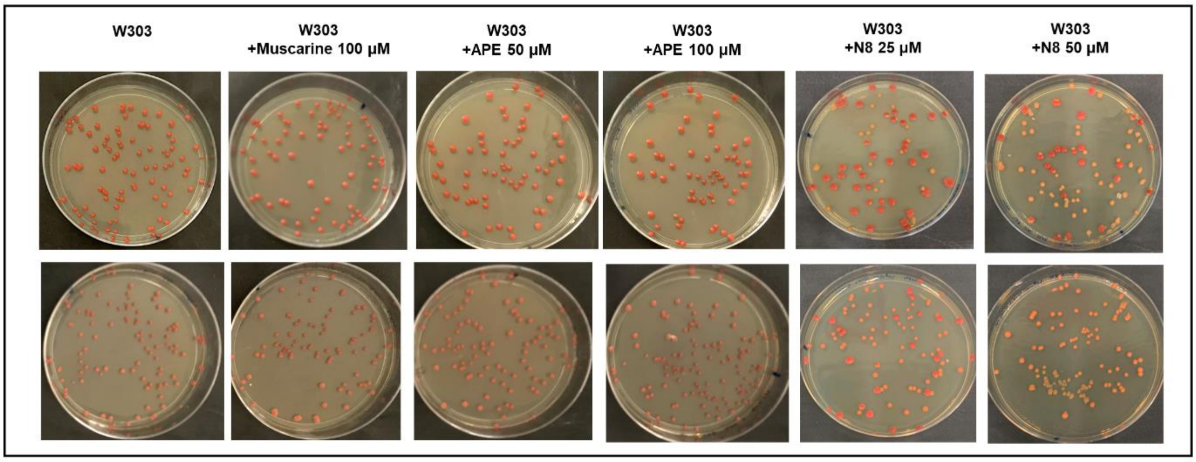

FIGURE S1: The muscarinic agonist N-8-Iper **(N8) induces an high rate of petites production in yeast. Pictures show the growth of *S. cerevisiae* treated with muscarinic agonists in complete medium (YPD).** Pictures were taken after 24 hours (upper pictures) and 48 hours (lower pictures) of growth. The agonist N-8-Iper respiration impairement is time and concentration dependent. Muscarine, the control, and APE have no effect on yeast cells mitocondrial function. The colours of the colonies facilitates the screening of colonies with a defect in respiration: the W303 wild type yeast colonies are red because the ade2- mutation results in the accumulation of the substrate of the Ade2, an enzyme of the adenine biosynthetic pathway. The formation of this red intermediate is produced into the mitochondria. If the mitochondria are not functional, the intermediate is not produced and the colonies result in white/brown colonies [31, 32].

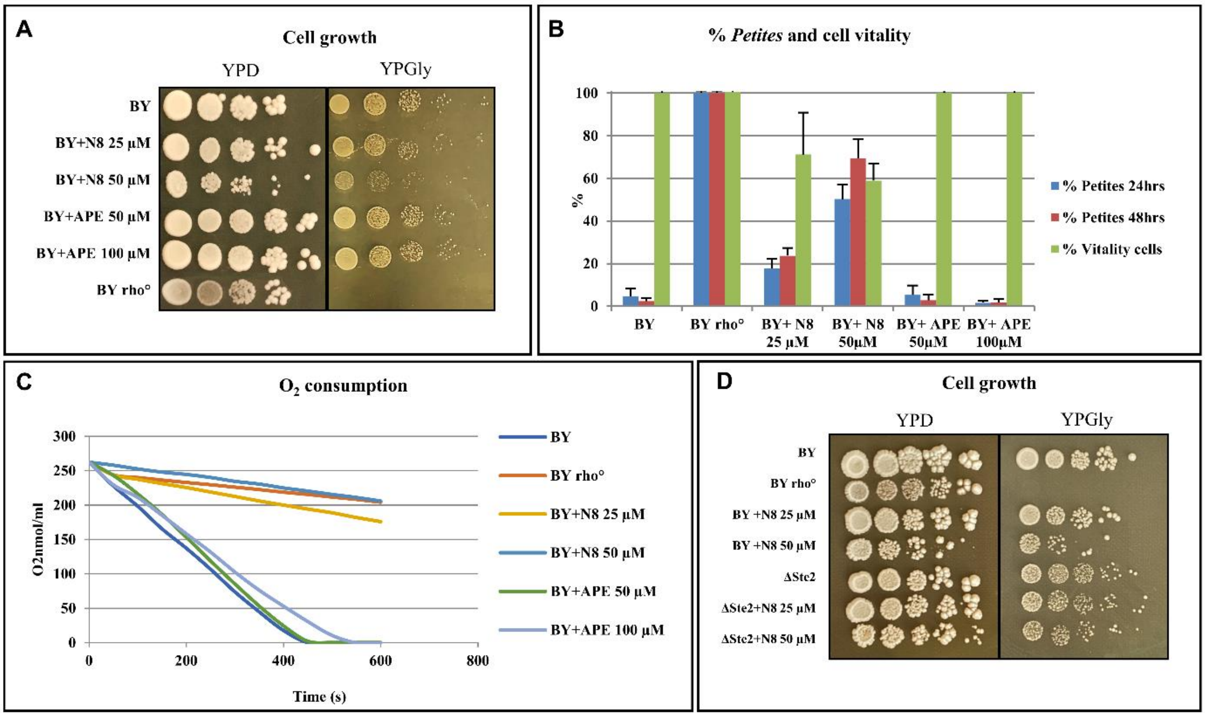

FIGURE S2: The muscarinic agonist N-8-Iper (N8) **induces a mitochondrial defect in the *S. cerevisiae* BY4741 wild type strain.** **(A)** Growth of BY4741 treated with muscarinic agonists in complete medium (YPD) and in a respiratory carbon source (YPGly), pictures taken after 24 hours of growth N-8-Iper imparirs the mitochondrial function while APE has no effect. **(B)** Percentage of petites production and vitality of wild type BY4741, BY4741 rho° grown in YPD medium and wild type BY4741 grown in YPD medium with agonists N-8-Iper (25 and 50 µM) and APE (50 and 100 µM). **(C)** The respiration rate of BY4741 yeast cells treated with N-8-Iper is impaired compared to the wild type strain, APE has no effect on the respiration rate. The oxygen consumption rate of BY4741, BY4741 rho° and BY4741 cells treated with agonists is expressed in O2nmol/ml. **(D)** Growth of BY4741 and ΔSte2 treated with muscarinic agonists in complete medium (YPD) and in a respiratory carbon source (YPGly), pictures taken after 24 hours of growth N-8-Iperimparirs the mitochondrial function.

REFERENCES

31. Bernardi G (**1979**). The petite mutation in yeast. **Trends Biochem Sci** 4(9): 197–201. doi: 10.1016/0968-0004(79)90079-3

32. Bharathi V, Girdhar A, Prasad A, Verma M, Taneja V, and Patel BK (**2016**). Use of ade1 and ade2 mutations for development of a versatile red/white colour assay of amyloid-induced oxidative stress in *Saccharomyces cerevisiae*. **Yeast** 33(12): 607–620. doi: 10.1002/yea.3209
